# Scarlet Fever—The Dangers of Convalescence

**Published:** 1887-10-29

**Authors:** 


### The Doctor's Pulpit.

### SCARLET FEVER—THE DANGERS OF CONVALESCENCE.

Many readers will remember the trouble and distress which were brought upon Professor Nettleship, of Oxford, and his family last year, in consequence of their having spent a part of their holiday in a rented farmhouse in which were some scarlet-fever convalescents. The learned Professor had engaged a house for the autumn at New Quay, and at the time appointed betook himself with his belongings to the place, anticipating no doubt a pleasant and invigorating holiday. The state of his mind may be imagined when one of his children began to show symptoms of scarlet fever ; and his feelings sharpened into indignation when he discovered that some members of the farmer's household had been in the "peeling" stage of the fever at the time of his entrance upon his temporary tenancy. A similar case, but attended with much more serious consequences, occurred during the present year. A lady with her young children went for their usual change of air to the seaside, and were received into a house where some members of the family were recovering from this fever. The lady, all unconscious of her danger, took possession of what proved to be a home of death. In a very few days almost all the members of her family were down with the disease, and such was the virulence of the attack that two of them speedily died. Other cases occur to the mind whilst writing, all of which emphasise the two points that scarlet fever is a highly infectious disease, and that the stage of greatest virulence is the convalescent period.

Everybody knows that what has just been stated is in accordance with universal experience. No practical person doubts the extreme risks run by those who from accident or necessity associate with persons who are desquamating or "peeling" after scarlatina. And yet such cases as those recorded could be multiplied a hundredfold. Constantly, in all parts of the kingdom, and in almost every social grade, we are witnessing a carelessness which is as dangerous as ignorance and as culpable as it is dangerous : a carelessness which swells the returns of mortality many times over, and brings mourning and lamentation into innumerable households. Scarlet-fever convalescents are infinitely more dangerous than mad dogs. Where one person dies from rabies a hundred die of scarlet fever, many of whom would never have had the disease at all if they had not come into contact with some patient in the dangerous stage. It is one of the constant difficulties of family practice to get people to believe in the necessity for continued isolation. They see the fever is over, that appetite and strength are returning, that fresh air is longed for, and in mere kindness of heart they give way to a child's request, and bring the time of separation to an abrupt close. Ten or twelve days added to the isolation period would not be injurious to the patient, and would in many cases mean safety to others instead of danger, or even disease and death.

Isolation should be complete for six or eight weeks. It is not necessary to confine the patient to his room all that time, unless it is impossible to keep him separated by any other means. If the kidneys be healthy, fresh air is highly desirable, both as a means of hastening desquamation and of restoring the wasted strength. To accomplish this object, scarlet-fever convalescent homes are urgently demanded, and especially for that large class who have no other prospect of change of air. But even in the case of well-to-do persons it is neither safe nor right that their scarlatinal patients should be taken to the abodes of private lodging-house keepers. They, too, should be kept completely isolated, either at a special convalescent home for paying patients, or at a private residence where separation and disinfection can be as thoroughly effected as in a hospital.

This complete isolation is a proceeding which demands much thought and care, both where patients are under the control of hospital authorities, and where they are in charge of their own relatives and the family medical attendant. Supposing removal to be resolved upon, in either case the patient, before leaving his room, should be anointed from head to foot with an oil or unguent with which some disinfectant has been mixed, for the obvious purpose of rendering the light feathery scales of the skin adherent. He should then be folded carefully in a large wrapper, which after being used can be well soaked in a disinfectant fluid. The ambulance should be at the door, and if it be practicable, as it generally is, the patient should not walk, but be carried to it. The ambulance itself must of course be disinfected ; and it should, wherever possible, be the only carriage used between the place of starting and the final destination of the patient. If, however, a railway, river, or cab journey is unavoidable, on no account should the public vehicle be made use of without due warning to the railway, steamship, or carriage company. They in their turn will select a special compartment, which will be duly disinfected before being again used by the public.

So urgent is this necessity for complete isolation that steps should be taken by the constituted authorities to make it compulsory. No person or family ought to have the right to endanger the lives of hundreds of fellow-beings at pleasure. The law does not permit a man to stalk around his neighbour's corn-stacks with a lighted torch. But he who permits a child who has had scarlatina to run about during the period of infection, or in any way to have access to others, may inflict far greater wrong and misery upon his fellows than he who sets a stackyard on fire.

With the majority of people, to point out the way of safety is to ensure their walking in it ; but there are always some ill-conditioned persons who will do nothing whatever in the interests of their neighbours unless they are compelled. The cases quoted at the beginning of this article are samples of what is frequently occurring in different parts of the country. Not only ought the law to insist upon perfect isolation for a sufficient length of time, but offenders should be punished with such severity as to ensure the observance of the statute. The farmer at New Quay was fined ten pounds in Professor Nettleship's case. Ten pounds was no compensation to the Professor for the ruining of his holiday and the grief and anxiety he had to endure. But what if his child had died? Could any possible amount of money have atoned at all for the loss? Let the public be instructed by all means, and where persuasion is adequate stronger measures reed not be adopted. But as Gibbon has said, " Persuasion is the resource of the feeble, and the feeble can seldom persuade." In cases of the kind under discussion the " strong arm of the law " should undoubtedly be called into requisition. If we legislate against poachers, tramps, and beggars, who merely cause a certain amount of annoyance and the loss of a little not very valuable property, it is surely of greater moment to put the law in motion on behalf of the health and lives of our children. Even as a matter of economy an epidemic of fever is a serious consideration. Look at the cost to the public of removing, maintaining, and medically supervising the hundreds of fever patients now under care for six or eight weeks. A single case of scarlet fever in the house of one of the wealthier classes can hardly cost less than twenty pounds for medical attendance, nursing, disinfecting, and all other necessary disbursements: it may cost very much more. On all grounds, therefore, of cost, of the public well-being, of private grief and anxiety, and of safety to the children, whose ignorance is their danger, all possible means should be adopted to completely isolate, and so render harmless, every case of scarlet fever until desquamation is entirely completed.

